# Task-Based Model Observer Assessment of A Partial Model-Based Iterative Reconstruction Algorithm in Thoracic Oncologic Multidetector CT

**DOI:** 10.1038/s41598-018-36045-4

**Published:** 2018-12-07

**Authors:** David C. Rotzinger, Damien Racine, Catherine Beigelman-Aubry, Khalid M. Alfudhili, Nathalie Keller, Pascal Monnin, Francis R. Verdun, Fabio Becce

**Affiliations:** 1 Department of Diagnostic and Interventional Radiology, Lausanne University Hospital, Rue du Bugnon 46, 1011 Lausanne, Switzerland; 2 Institute of Radiation Physics, Lausanne University Hospital, Rue du Grand-Pré 1, 1007 Lausanne, Switzerland

## Abstract

To investigate the impact of a partial model-based iterative reconstruction (ASiR-V) on image quality in thoracic oncologic multidetector computed tomography (MDCT), using human and mathematical model observers. Twenty cancer patients examined with regular-dose thoracic-abdominal-pelvic MDCT were retrospectively included. Thoracic images reconstructed using a sharp kernel and filtered back-projection (reference) or ASiR-V (0–100%, 20% increments; follow-up) were analysed by three thoracic radiologists. Advanced quantitative physical metrics, including detectability indexes of simulated 4-mm-diameter solid non-calcified nodules and ground-glass opacities, were computed at regular and reduced doses using a custom-designed phantom. All three radiologists preferred higher ASiR-V levels (best = 80%). Increasing ASiR-V substantially decreased noise magnitude, with slight changes in noise texture. For high-contrast objects, changing the ASiR-V level had no major effect on spatial resolution; whereas for lower-contrast objects, increasing ASiR-V substantially decreased spatial resolution, more markedly at reduced dose. For both high- and lower-contrast pulmonary lesions, detectability remained excellent, regardless of ASiR-V and dose levels, and increased significantly with increasing ASiR-V levels (all p < 0.001). While high ASiR-V levels (80%) are recommended to detect solid non-calcified nodules and ground-glass opacities in regular-dose thoracic oncologic MDCT, care must be taken because, for lower-contrast pulmonary lesions, high ASiR-V levels slightly change noise texture and substantially decrease spatial resolution, more markedly at reduced dose.

## Introduction

Multidetector computed tomography (MDCT) is the workhorse in cancer imaging for initial diagnosis, staging, restaging after neoadjuvant therapy, as well as monitoring response to treatment, including the assessment of pulmonary metastases^1–3^. Despite major efforts to reduce radiation dose, combined thoracic-abdominal-pelvic oncologic MDCT remains a substantial source of exposure and therefore a concern, particularly in younger patients requiring repeated scans over lengthy follow-up periods^4,5^.

Iterative reconstruction (IR) techniques are the latest of numerous instruments developed by manufacturers over the past decade to enable MDCT dose savings^6–8^. Although their successful implementation in thoracic MDCT has helped to substantially reduce dose (depending on the initial dose level and clinical setting) without compromising image quality compared to the conventional filtered back-projection (FBP) algorithm^9,10^, relatively few studies have thoroughly assessed the impact of IR algorithms on the analysis of the fine structures of the pulmonary parenchyma, which require the use of sharp (high spatial resolution) reconstruction kernels^11–17^. Furthermore, even fewer investigations have properly evaluated the effects of latest-generation partial model-based IRs, such as adaptive statistical IR-V (ASiR-V; GE Healthcare, Waukesha, WI, USA)^18,19^, advanced model-based IR (ADMIRE; Siemens Healthineers, Forchheim, Germany)^13,20^, and iterative model reconstruction (IMR; Philips Healthcare, Best, The Netherlands)^11,17,21,22^, on image quality. Moreover, and most importantly, the vast majority of previous studies have ignored the non-linearity of commercially available IR algorithms, which make image noise non-stationary and thus highly spatial-dependent, as well as spatial resolution contrast-dependent^23–27^. These previous investigations have therefore used conventional (e.g. contrast-to-noise ratio, CNR) and/or more advanced Fourier-based (e.g. modulation transfer function, MTF) image quality metrics ill-suited to IR algorithms, such as the MTF rather than the object-specific target transfer function (TTF) for assessing contrast-dependent spatial resolution.

Because the inappropriate use of technological advances in MDCT may have detrimental effects on image quality and diagnostic performance, and ultimately patient management, we aimed to investigate the impact of ASiR-V on subjective and objective image quality in thoracic oncologic MDCT, using appropriate objective physical metrics and a task-based approach. Our secondary objectives were to determine the optimal ASiR-V level for pulmonary parenchyma analysis at regular dose, and explore the effects of additional dose reduction on task-based objective image quality.

## Methods

### Study Design and Patients

This single-centre retrospective longitudinal study was approved by the institutional ethics committee of Lausanne University Hospital (CER-VD, protocol 847/16), with waiver of informed consent.

We selected 20 consecutive patients (11 men, 9 women; mean age 63.8 ± 10.7 years; range 45–81 years) examined in March 2015 with routine combined thoracic-abdominal-pelvic MDCT as part of their cancer surveillance (gastrointestinal, n = 7; thoracic, n = 5; genitourinary, n = 4; other cancers, n = 4). Inclusion criteria were: patients ≥18 years of age, availability of a reference scan performed on one of the institution’s earlier MDCT system from the same manufacturer, no contraindication to intravenous administration of iodinated contrast medium, haemodynamic stability, ability to hold their breath for the duration of the follow-up MDCT scan, and no motion artefacts on either of the two scans. The mean time interval between the reference and follow-up scans was 10.3 ± 4.1 months (range, 5–14 months).

### MDCT Protocols

**Figure 1:**
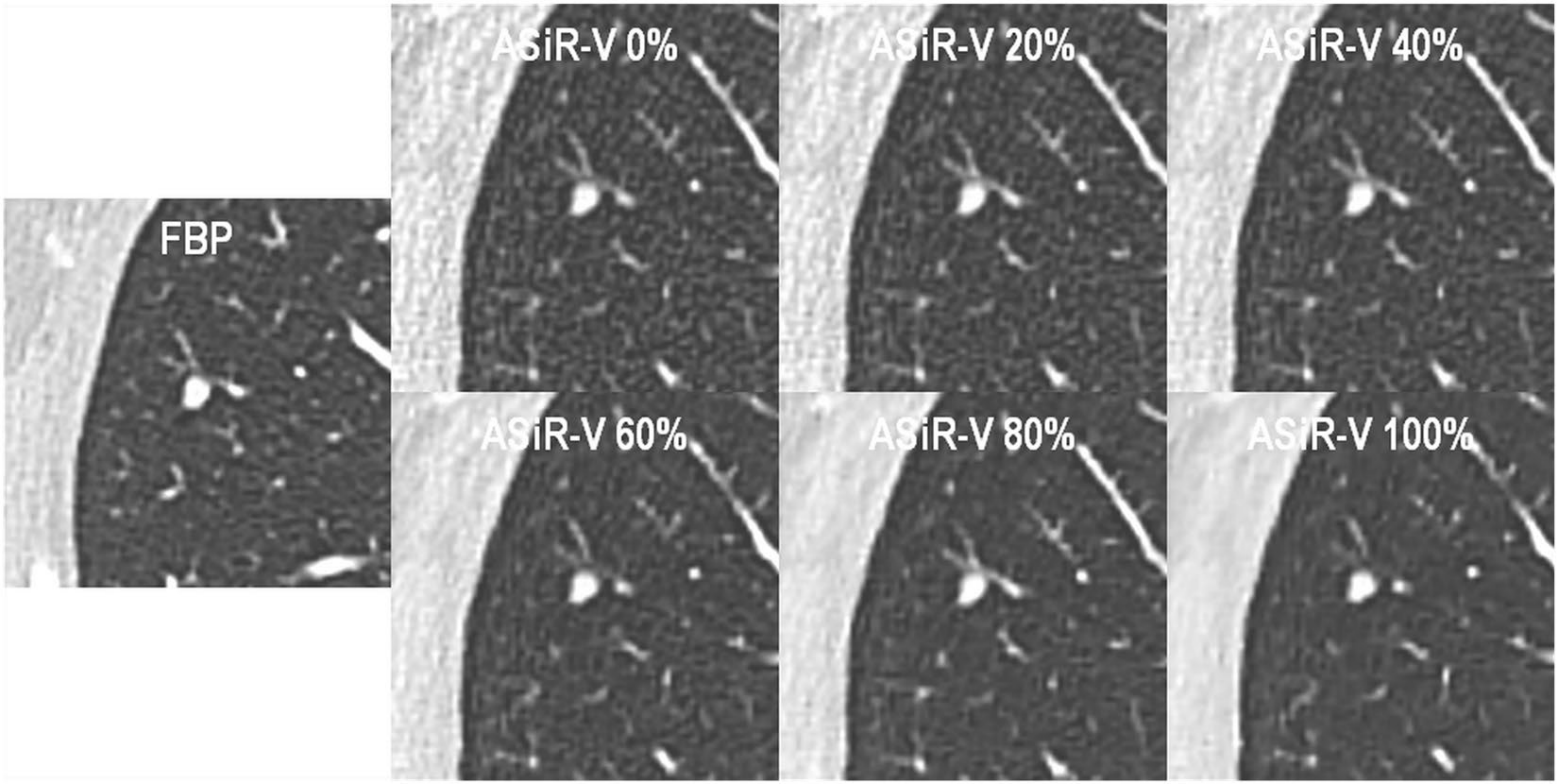
59-year-old man with metastatic melanoma skin cancer. Axial contrast-enhanced thoracic MDCT images (lung kernel; window centre/width, −600/1600 HU) show a solid non-calcified pulmonary nodule in the right upper lobe. Images of reference and follow-up scans were reconstructed using FBP and increasing ASiR-V levels (0–100%, in 20% increments), respectively. Note the decrease in noise magnitude and change in noise texture, as well as decrease in spatial resolution for fine lower-contrast pulmonary structures with increasing ASiR-V levels.

MDCT scans were performed on either a 64-detector row system (LightSpeed VCT, GE Healthcare) for reference scans, or a 256-detector row system (Revolution CT, GE Healthcare) for follow-up scans, using the helical acquisition mode and following intravenous administration of iodinated contrast medium (Accupaque 300, GE Healthcare; 1 mL/kg of body weight +30 mL, at a flow rate of 3 mL/s, followed by 40 mL of saline flush). Patients were positioned supine with their arms raised above the head, and instructed to hold their breath at full inspiration during the scan. Parameters for data acquisition and image reconstruction on the two MDCT systems are detailed in Table 1. We reduced the projected dose level on the 256-MDCT system by 21% owing to the 25% reduction in electronic noise offered by its detectors (Gemstone Clarity Detector, GE Healthcare), as per the manufacturer’s data. Although the 64-MDCT system would have allowed the use of first-generation hybrid ASiR algorithm for reference scans^28^, we were reluctant to implement IRs before having investigated them, and the latter could not be applied retroactively because raw data were no longer available at the time of the study. Therefore, only follow-up scans were reconstructed using the following increasing levels of ASiR-V: 0%, 20%, 40%, 60%, 80%, and 100% (Fig. 1).

**Table 1:** Parameters for MDCT data acquisition and image reconstruction.

MDCT system	Reference MDCT scan	Follow-up MDCT scan	
	LightSpeed VCT	Revolution CT	
Radiation dose level	Regular	Regular	Reduced^*^
**Data acquisition**			
Tube potential (kVp)	120	120	120
Tube current (mA)	400	400	90
Automatic exposure control (noise index)	Combined xyz-axis (25)	Combined xyz-axis (12.5)	Combined xyz-axis (12.5)
Gantry revolution time (s)	0.6	0.5	0.5
Beam collimation (mm)	64 × 0.625	128 × 0.625	128 × 0.625
Beam pitch	1.375	0.992	0.992
Scan field of view (cm)	32 × 32	32 × 32	32 × 32
**Image reconstruction**			
Display field of view (cm)	28 × 28–40 × 40	29 × 29–41 × 41	32 × 32
Section thickness (mm)	1.25	1.25	1.25
Section overlap (mm)	1	1	1
Kernel	Lung	Lung	Lung
Algorithm	FBP	ASiR-V 0%, 20%, 40%, 60%, 80%, and 100%	ASiR-V 0%, 20%, 40%, 60%, 80%, and 100%

ASiR-V = adaptive statistical iterative reconstruction-V, CTDI_vol_ = volume computed tomography dose index, FBP = filtered back-projection, MDCT = multidetector computed tomography, *phantom only.

#### Radiation Dose Estimates

Volume CT dose indexes (CTDI_vol_) of the thoracic region were retrieved from the radiation-dose structured reports for both reference and follow-up MDCT scans. Size-specific dose estimates (SSDE) were then calculated using the following formula:

$${\rm{SSDE}}={f}_{{\rm{size}}}^{32{\rm{D}}}\times {{\rm{CTDI}}}_{{\rm{vol}}}^{32{\rm{D}}}$$

where ${f}_{{\rm{size}}}^{32{\rm{D}}}$ is the conversion factor as a function of the patient’s effective diameter^29^. Patients’ effective diameters were determined from the maximum anteroposterior and lateral dimensions on a mid-thoracic axial MDCT image. We did not consider dose-length products due to interpatient protocol variability, with varying phase numbers on the abdomen and pelvis.

### Image Analysis

All MDCT images were analysed on a picture archiving and communication system workstation (Vue PACS; Carestream Health, Rochester, NY, USA) by four independent observers. For the study purposes, we only reviewed thoracic images reconstructed using the sharp kernel dedicated to pulmonary parenchyma analysis (lung, GE Healthcare), and displayed with the default lung window centre (−600 HU) and width (1600 HU) settings.

#### Conventional Image Quality Metrics

Quantitative Analysis: A radiology resident performed all measurements, and repeated them after a 3-month interval to assess reliability. First, axial images of the reference (reconstructed using FBP) and follow-up (reconstructed using ASiR-V 0%) MDCT scans were registered. Then, two circular 1-cm^2^ regions of interest (ROI) were drawn into the most homogeneous regions of tracheal air, just above the carina, and erector spinae muscles on images of the reference scan. ROIs were subsequently copied and pasted on the corresponding images of the follow-up scan, first on ASiR-V 0%, followed by the five increasing ASiR-V levels up to 100%. Measurements were obtained for three consecutive sections, and mean values (mean CT numbers and standard deviations (SD), in HU) calculated. Image noise was defined as the SD of CT numbers within ROIs in tracheal air, whereas the signal-to-noise ratio (SNR) and CNR were calculated as follows:

$${\rm{SNR}}=\frac{|{\rm{mean}}\,{\rm{CT}}\,{{\rm{number}}}_{{\rm{air}}}|}{{{\rm{SD}}}_{{\rm{air}}}}$$

$${\rm{CNR}}=\frac{|{\rm{mean}}\,{\rm{CT}}\,{{\rm{number}}}_{{\rm{air}}}-{\rm{mean}}\,{\rm{CT}}\,{{\rm{number}}}_{{\rm{muscle}}}|}{\sqrt{\frac{1}{2}({{\rm{SD}}}_{{\rm{air}}}^{2}+{{\rm{SD}}}_{{\rm{muscle}}}^{2})}}$$

Qualitative Analysis: First, in order to become accustomed to the appearance of images reconstructed using ASiR-V and agree on the grading system detailed below, a senior and two junior thoracic radiologists (with 25 and 5 years of experience, respectively) jointly reviewed five test cases, the images of which were acquired and reconstructed in the same way as for the study but which were not included. They subsequently assessed all MDCT scans independently and blinded to the image reconstruction algorithm. As a first step, observers were asked to determine their preferred ASiR-V level among the various reconstructions of the follow-up scans, ranking them from best to worst using a 6-point ordinal scale (6 = best, 1 = worst). Their ratings were based on the European guidelines on quality criteria for chest CT, more specifically the visually sharp reproduction of lung parenchyma, secondary pulmonary lobular structures, pulmonary vessels, bronchi, and pleural borders^30^. In a second step, observers evaluated the overall diagnostic image quality of FBP images of reference scans and the best-rated ASiR-V reconstruction of follow-up scans (based on the average score of independent assessments in step 1) using a 4-point scale (4 = exemplary, 3 = diagnostic, 2 = limited, 1 = non-diagnostic, as per the Radiologic Society of North America radiology lexicon). They also assessed the following IR-related specific pulmonary MDCT features and artefacts using a 4-point scale (4 = severe, 3 = moderate, 2 = mild, 1 = none): distorted appearance of the fine structures of secondary pulmonary lobules, blurring of vascular and/or bronchial borders, and hypoattenuating bands at tissue interfaces^31,32^.

#### Fourier-Based Image Quality Metrics

We scanned a custom-designed 25-cm-diameter cylindrical phantom on the same two MDCT systems using the same settings for data acquisition and image reconstruction as for patients (Table 1). The phantom was further rescanned at reduced dose (projected CTDI_vol_, 3 mGy) on the 256-MDCT system by decreasing only the tube current (90 mA; Table 1). The 5-mm-thick phantom shell was made of polymethylmethacrylate (PMMA) and its centre contained 10-cm-diameter cylindrical inserts made of polytetrafluoroethylene (PTFE; mean CT number, 1080 HU at 120 kVp) and PMMA (mean CT number, 120 HU at 120 kVp), both surrounded by water so that the attenuation differences (|ΔHU|) between PTFE-water and PMMA-water at 120 kVp were similar to those found in patients in the thoracic region: approximately 1000 HU between solid non-calcified pulmonary nodules and lung parenchyma, and 150 HU between ground-glass opacities and lung parenchyma, respectively^33–35^.

Noise Power Spectrum: Image noise was quantified and characterised by computing noise power spectra (NPS) according to the recommendations from the International Commission on Radiation Units and Measurements Reports 54 and 87, and the method described by Miéville *et al*.^36–38^. Measurements were performed on 40 consecutive axial MDCT images taken at the phantom extremity and containing only water (no central insert). On each section, a square-shaped ROI of 170 × 170 pixels was automatically extracted from the centre of the image and 2D NPS were obtained, and then radially averaged to generate 1D NPS, using user-defined Matlab scripts (The MathWorks, Natick, MA, USA).

Target Transfer Function: In-plane contrast-dependent spatial resolution was quantified and characterised by computing TTFs using Matlab scripts (The MathWorks), as described in detail by Ott *et al*.^34^. Measurements were performed on 50 and 575 axial MDCT images for regular- and reduced-dose protocols, respectively. On each section, a square-shaped ROI of 170 × 170 pixels encompassing the central insert and its interface with the surrounding water was automatically extracted from the image. TTFs were calculated from the edge spread function profiles. Raw data of the edge spread functions were first fitted and analytically differentiated to generate line spread functions. 2D TTFs were obtained by applying a Fourier transform on the line spread functions, which were then radially averaged and normalised to 1 at zero frequency to yield 1D TTFs.

#### Task-Based Image Quality Assessment

Nonprewhitening With Eye Filter Model Observer: To account for and quantify the combined effects of contrast, noise, and spatial resolution on objective MDCT image quality, we computed detectability indexes (d′) using a nonprewhitening with eye filter (NPWE) model observer. The NPWE is a mathematical model observer first developed by Burgess^39^ and recently updated for IR algorithms by Ott *et al*.^34^. Detectability indexes of simulated 4-mm-diameter circular solid non-calcified nodules (PTFE) and ground-glass opacities (PMMA) were calculated as follows:

$$d\text{'}=\frac{\sqrt{2{\rm{\pi }}}|{\rm{\Delta }}\mathrm{HU}|{\int }_{0}^{{{\rm{f}}}_{{\rm{Ny}}}}{{\rm{S}}}^{2}({\rm{f}}){{\rm{TTF}}}^{2}({\rm{f}}){{\rm{VTF}}}^{2}({\rm{f}}){\rm{fdf}}}{\sqrt{{\int }_{0}^{{{\rm{f}}}_{{\rm{Ny}}}}{{\rm{S}}}^{2}({\rm{f}}){{\rm{TTF}}}^{2}({\rm{f}}){\rm{NPS}}({\rm{f}}){{\rm{VTF}}}^{4}({\rm{f}}){\rm{fdf}}}}$$

where |ΔHU| is the absolute attenuation difference between PTFE-water and PMMA-water, respectively, f the radial spatial frequency, and f_Ny_ the Nyquist frequency of the image, S the Fourier transform of the input signal (in our case, ${\rm{S}}=\frac{{\rm{r}}}{{\rm{f}}}{{\rm{J}}}_{1}(2{\rm{\pi }}\mathrm{rf})$, with r the radius of the simulated disk and J_1_ a Bessel function of the first kind), and VTF the visual transfer function of the human eye^34^.

### Statistical Analysis

We analysed data using IBM SPSS Statistics (IBM, Armonk, NY, USA) and Matlab (The MathWorks). Unless otherwise specified, data are presented as mean ± SD. Continuous and categorical (ordinal) variables were compared using the Kruskal-Wallis (or one-way analysis of variance in the case of normal distribution according to the Shapiro-Wilk test) and Mann-Whitney U tests, where appropriate. Intraobserver reliability of quantitative measurements was assessed by calculating intraclass correlation coefficients (ICC), and interpreted as follows: <0.40, poor; 0.40–0.59, fair; 0.60–0.74, good; ≥0.75, excellent. Interobserver agreement for qualitative ratings was evaluated using weighted kappa coefficients, and interpreted as follows: <0.01, poor; 0.01–0.20, slight; 0.21–0.40, fair; 0.41–0.60, moderate; 0.61–0.80, substantial; ≥0.81, excellent. Areas under the receiver operating characteristic curve (AUC) were interpreted as follows: <0.71, poor; 0.71–0.80, fair; 0.81–0.90, good; ≥0.91, excellent. P-values < 0.05 were considered statistically significant, and adjusted (Dunn-Bonferroni method) for multiple post-hoc (Mann-Whitney U or Tukey tests, respectively) pairwise comparisons, where appropriate.

## RESULTS

### Radiation Dose Estimates

As per the study design, CTDIs_vol_ (7.5 ± 1.6 mGy) and SSDEs (9.4 ± 1.9 mGy) of follow-up MDCT scans were both significantly decreased by 22% (p = 0.004) and 21% (p < 0.001), respectively, compared with reference scans (9.6 ± 2.2 and 11.9 ± 1.9 mGy, respectively). Patients’ effective diameters did not change significantly between reference and follow-up scans (p = 0.871).

### Conventional Image Quality

#### Quantitative Analysis

The results of the quantitative analysis are reported in Table 2. Image noise was comparable between FBP images of reference MDCT scans and ASiR-V reconstructions of follow-up scans at 0%, 20%, and 40% (all p = 1), while taking into account both the reduced dose level and decreased electronic noise with the 256-MDCT system. Likewise, SNR and CNR were both comparable between FBP and 0%, 20%, and 40% ASiR-V reconstructions at 0%, 20%, and 40% (all p ≥ 0.672).

**Table 2:** Image noise, SNR, and CNR for reference and follow-up MDCT scans as a function of image reconstruction algorithms.

Reconstruction algorithm	Reference MDCT scan	Follow-up MDCT scan						P-value
	FBP	ASiR-V 0%	ASiR-V 20%	ASiR-V 40%	ASiR-V 60%	ASiR-V 80%	ASiR-V 100%	
Noise	66.4 ± 17.4	71.1 ± 8.6	63.9 ± 7.4	57.1 ± 7.8	50.0 ± 7.3	43.1 ± 6.8	35.9 ± 7.3	<0.001
SNR (air)	15.7 ± 5.4	13.9 ± 1.9	15.4 ± 2.1	17.5 ± 2.6	20.2 ± 3.3	23.8 ± 4.4	29.1 ± 6.7	<0.001
CNR (air-muscle)	2.5 ± 1.2	2.1 ± 0.4	2.5 ± 0.4	3.1 ± 0.6	3.9 ± 0.8	5.2 ± 1.3	7.5 ± 2.4	<0.001

Data are presented as mean ± standard deviation. ASiR-V = adaptive statistical iterative reconstruction-V, CNR = contrast-to-noise ratio, FBP = filtered back-projection, MDCT = multidetector computed tomography, SNR = signal-to-noise ratio.

On follow-up scans, noise decreased significantly, whereas SNR and CNR both increased significantly with increasing ASiR-V levels (all p < 0.001). Post-hoc pairwise comparisons revealed significant decreases in noise between 0% and 60% or higher ASiR-V (all p < 0.001), as well as for each 40% increment (upward and downward) starting from 20% ASiR-V (all p ≤ 0.026). In terms of SNR and CNR, differences were significant for each 40% increment starting from 0% ASiR-V (all p ≤ 0.049), except trends between 0% and 40% ASiR-V (p = 0.096), and 60% and 100% ASiR-V (p = 0.076) for SNR.

Intraobserver reliability was excellent for noise (all ICCs ≥ 0.905), SNR (all ICCs ≥ 0.787), and CNR (all ICCs ≥ 0.767), regardless of the image reconstruction algorithm.

#### Qualitative Analysis

**Figure 2:**
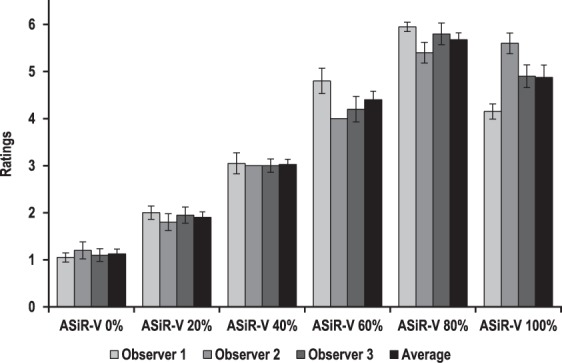
Subjective preferred ASiR-V levels (6 = best, 1 = worst) for the senior (observer 1) and two junior (observers 2 and 3) thoracic radiologists independently, and on average. All three observers significantly preferred higher ASiR-V levels, with average ratings peaking at 80% ASiR-V. Error bars represent 95% confidence intervals.

The main findings of the qualitative analysis are summarised in Fig. 2 and Table 3. All three observers preferred higher ASiR-V levels as their ratings increased significantly with the increase in ASiR-V level, up to 80% for the senior and one junior (observers 1 and 3, p < 0.001) and 100% for the second junior (observer 2, p < 0.001) thoracic radiologists, respectively (Fig. 2). Average qualitative scores also increased significantly to peak at 80% ASiR-V (p < 0.001). Post-hoc pairwise comparisons (for all observers and average scores) revealed significant differences in scores for all 20% ASiR-V increment (upward and downward) (all p ≤ 0.007), except between 80% and 100% ASiR-V (p = 1) for observer 2. Interobserver agreement was substantial (weighted kappa = 0.691–0.798).

**Table 3:** Subjective image quality scores for IR-related specific pulmonary MDCT features and artefacts.

		Reference MDCT scan	Follow-up MDCT scan	P-value
Distorted appearance of fine structures of secondary pulmonary lobules	Observer 1	1.0 ± 0.0	2.1 ± 0.2	<0.001
	Observer 2	1.4 ± 0.6	2.0 ± 0.8	0.017
	Observer 3	1.2 ± 0.4	1.8 ± 0.4	<0.001
Blurring of vascular borders	Observer 1	1.0 ± 0.0	1.1 ± 0.2	0.799
	Observer 2	1.4 ± 0.5	2.0 ± 0.8	0.030
	Observer 3	1.2 ± 0.4	1.3 ± 0.5	0.270
Blurring of bronchial borders	Observer 1	1.3 ± 0.5	1.5 ± 0.5	0.289
	Observer 2	1.4 ± 0.5	2.0 ± 0.7	0.007
	Observer 3	1.1 ± 0.3	1.3 ± 0.4	0.226
Hypoattenuating bands at tissue interfaces	Observer 1	1.9 ± 0.4	2.3 ± 0.5	0.056
	Observer 2	2.2 ± 0.4	2.4 ± 0.6	0.265
	Observer 3	2.0 ± 0.4	2.1 ± 0.3	0.195

Data are presented as mean ± standard deviation. MDCT = multidetector computed tomography.

When comparing FBP images of reference MDCT scans with the best-rated ASiR-V reconstruction of follow-up scans in terms of overall diagnostic image quality, we found a significant decrease for observer 1 (3.5 ± 0.6 and 3.0 ± 0.4, respectively; p = 0.004), as opposed to a non-significant increase (i.e. statistically comparable) for observer 2 (3.0 ± 0.5 and 3.2 ± 0.7, respectively; p = 0.396), and non-significant decrease for observer 3 (3.4 ± 0.5 and 3.3 ± 0.4, respectively; p = 0.507). However, average scores remained diagnostic for all three observers. Interobserver agreement ranged from slight to moderate for FBP (weighted kappa = 0.063–0.455), and slight to fair for ASiR-V (weighted kappa = 0.040–0.200) images.

Finally, the scores for IR-related specific pulmonary MDCT features and artefacts are presented in Table 3. All three observers perceived significantly more artefacts on ASiR-V images (Table 3). In particular, the distorted appearance of the fine structures of secondary pulmonary lobules was slightly though significantly emphasised for observer 1 (p < 0.001), whereas observer 2 also noted slight increases in blurring of both vascular and bronchial borders (p = 0.030 and 0.007, respectively).

### Fourier-Based Image Quality

#### Noise Power Spectra

**Figure 3:**
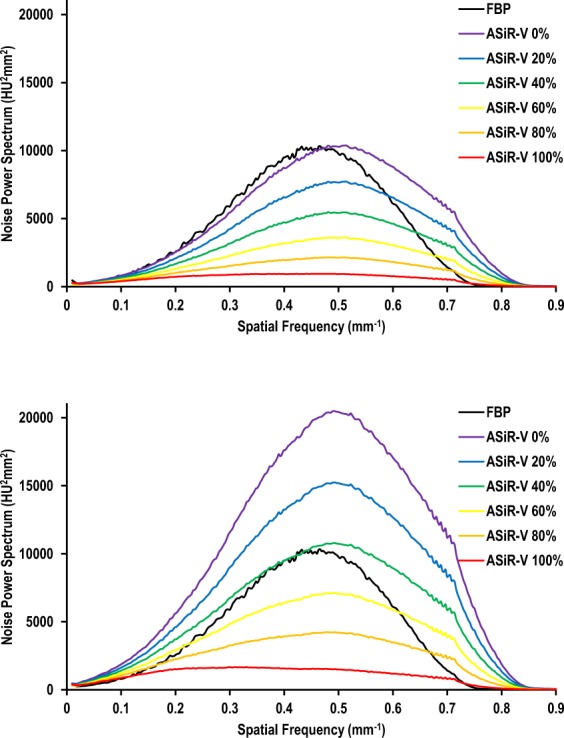
Noise power spectra as a function of the image reconstruction algorithm at regular (**a**) and reduced (**b**) dose levels. Increasing the ASiR-V level substantially decreased noise magnitude (i.e. area under the spectral curve), with slight changes in noise texture (i.e. spatial frequency content) compared with FBP, regardless of dose.

The impact of ASiR-V algorithm on image noise as a function of the radiation dose level is illustrated in Fig. 3. At regular dose, noise magnitude (i.e. area under the spectral curve) was comparable between FBP images of reference MDCT scans and 0% ASiR-V reconstructions of follow-up scans, while considering both the reduced dose level and decreased electronic noise with the 256-MDCT system (Fig. 3a). However, noise texture (i.e. spatial frequency content, higher spatial frequencies implying finer noise texture, whereas lower frequencies indicating coarser or grainier texture) was slightly changed as spectral curve centroids were shifted toward higher spatial frequencies (from 11.3% to 0.8% difference with FBP for 0% and 100% ASiR-V, respectively). Increasing the ASiR-V level substantially decreased noise magnitude. When comparing regular-dose FBP with reduced-dose ASiR-V, 40% ASiR-V was needed to compensate for the additional dose reduction (Fig. 3b). Changing the ASiR-V level at reduced dose had similar effects on both noise magnitude and texture (from 9.6% to −3.4% difference with FBP for 0% and 100% ASiR-V, respectively) as at regular dose.

#### Target Transfer Functions

**Figure 4:**
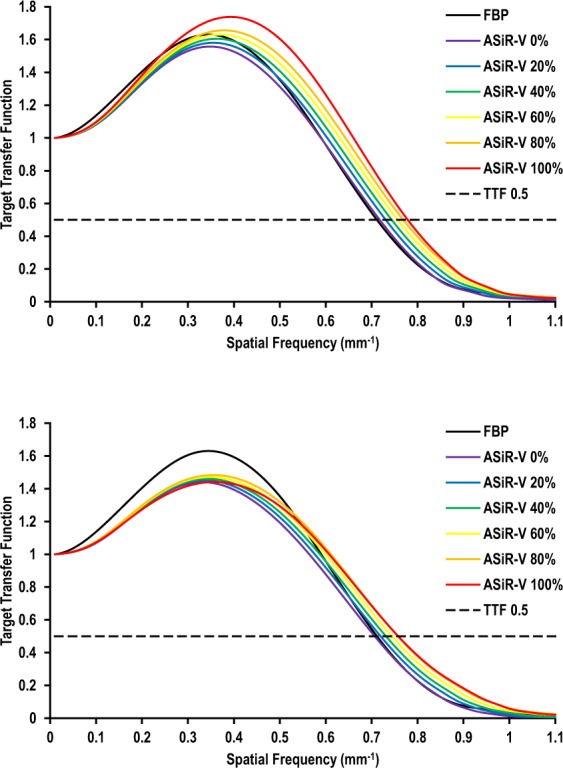
Target transfer functions as a function of the image reconstruction algorithm for high-contrast objects at regular (**a**) and reduced (**b**) dose levels. High-contrast spatial resolution was comparable between FBP and ASiR-V, regardless of dose. Increasing the ASiR-V level had no substantial impact on spatial resolution, with slight differences in TTF at 0.5 (dashed line), regardless of dose.

**Figure 5:**
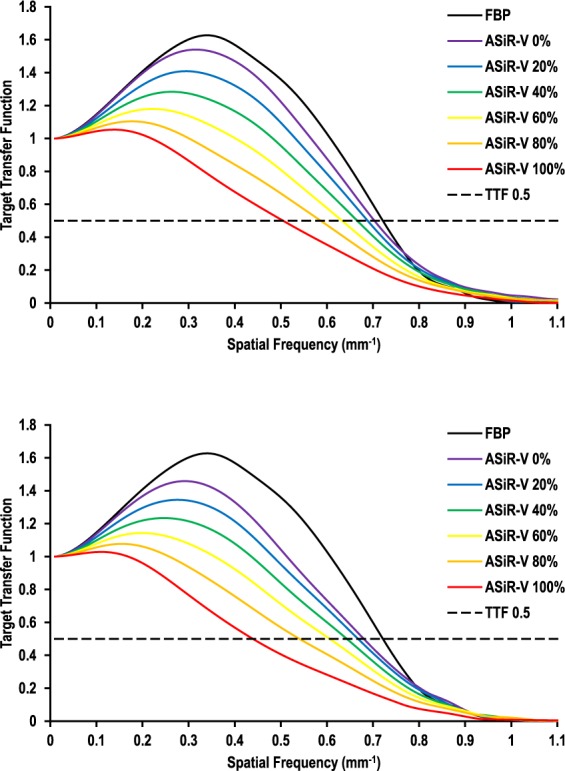
Target transfer functions as a function of the image reconstruction algorithm for lower-contrast objects at regular (**a**) and reduced (**b**) dose levels. Compared with FBP, increasing the ASiR-V level substantially decreased spatial resolution at regular dose (**a**), and more markedly at reduced dose (**b**). The dashed line represents the TTF at 0.5.

Figures 4 and 5 depict the impact of ASiR-V on spatial resolution as a function of contrast and dose levels, respectively. For high-contrast objects (materials), spatial resolution was comparable between FBP and ASiR-V, regardless of dose (Fig. 4). Changing the ASiR-V level had no substantial effect on spatial resolution, with slight differences in TTF at 0.5 (from 0.8% to 9.5% difference with FBP for 0% and 100% ASiR-V, respectively). However, for lower-contrast objects, increasing the ASiR-V level substantially decreased spatial resolution at regular dose (from −1.2% to −29.3% difference with FBP for 0% and 100% ASiR-V, respectively) (Fig. 5a), and more markedly at reduced dose (from −4.4% to −38.4% difference with FBP for 0% and 100% ASiR-V, respectively) (Fig. 5b).

### Task-Based Image Quality

#### Nonprewhitening With Eye Filter Model Observer

**Figure 6:**
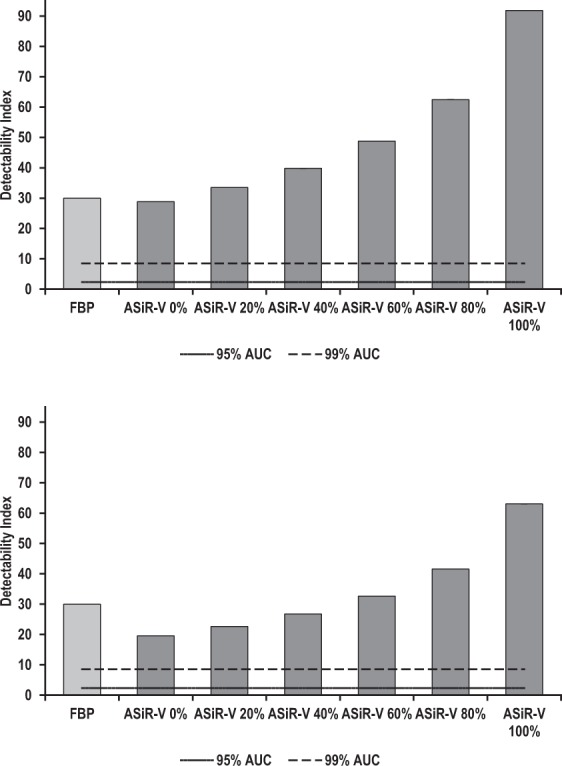
Detectability indexes (d′) of simulated 4-mm-diameter solid non-calcified nodules as a function of the image reconstruction algorithm at regular (**a**) and reduced (**b**) dose levels. At regular dose, mean (95% confidence intervals) d’ ranged from 30.0 (29.9, 30.1) to 28.8 (28.8, 28.9) and 91.9 (91.6, 92.2) for FBP and 0% to 100% ASiR-V, respectively; whereas at reduced dose, mean (95% confidence intervals) d′ varied from 30.0 (29.9, 30.1) to 19.5 (19.5, 19.6) and 63.0 (62.9, 63.2) for FBP and 0% to 100% ASiR-V, respectively. Compared with FBP, detectability remained excellent (AUC ≥ 0.91), regardless of ASiR-V and dose levels. Detectability increased significantly with increasing ASiR-V levels to peak at 100% ASiR-V. Error bars represent 95% confidence intervals; they overlap with vertical bars. The solid and dashed lines represent 95% and 99% AUC, respectively.

**Figure 7:**
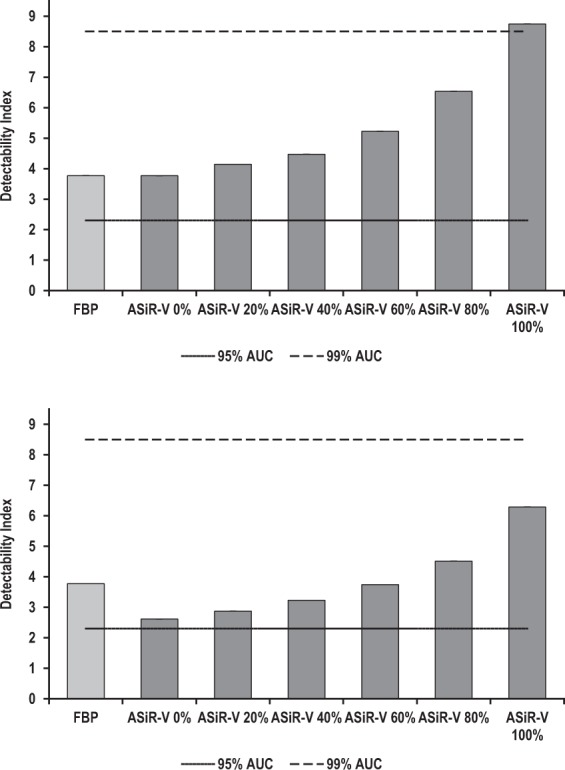
Detectability indexes (d′) of simulated 4-mm-diameter ground-glass opacities as a function of the image reconstruction algorithm at regular (**a**) and reduced (**b**) dose levels. At regular dose, mean (95% confidence intervals) d’ ranged from 3.8 (3.8, 3.8) to 3.8 (3.8, 3.8) and 8.8 (8.7, 8.8) for FBP and 0% to 100% ASiR-V, respectively; whereas at reduced dose, mean (95% confidence intervals) d′ varied from 3.8 (3.8, 3.8) to 2.6 (2.6, 2.6) and 6.3 (6.2, 6.4) for FBP and 0% to 100% ASiR-V, respectively. Compared with FBP, detectability remained excellent (AUC ≥ 0.91) yet slightly lower than for solid non-calcified nodules, regardless of ASiR-V and dose levels. Detectability increased significantly with increasing ASiR-V levels to peak at 100% ASiR-V. Error bars represent 95% confidence intervals; they overlap with vertical bars. The solid and dashed lines represent 95% and 99% AUC, respectively.

Detectability indexes of simulated 4-mm-diameter solid non-calcified nodules and ground-glass opacities are shown in Figs 6 and 7, respectively. For high-contrast lesions (nodules), d’ remained excellent (>99% AUC), irrespective of ASiR-V and dose levels, and increased significantly with the increase in ASiR-V level to peak at 100% ASiR-V (all p < 0.001) (Fig. 6a,b). For lower-contrast lesions (opacities), the same effects of ASiR-V and dose levels were observed (all p < 0.001), with excellent yet slightly lower d’ (>95% AUC for reduced-dose 0% ASiR-V) (Fig. 7a,b).

## Discussion

The present study shows that increasing the ASiR-V level in regular-dose thoracic oncologic MDCT substantially decreased noise magnitude, with only slight changes in noise texture, at both investigated dose levels. We also found that the spatial resolution properties strongly depended on the object-to-background contrast level when using ASiR-V: for high-contrast objects (|ΔHU| ≈ 1000 HU), changing the ASiR-V level had no major effect on spatial resolution, regardless of dose; whereas for lower-contrast objects (|ΔHU| ≈ 150 HU), increasing the ASiR-V level substantially decreased spatial resolution, more markedly at reduced dose. Due to extra smoothing, the ability of ASiR-V algorithm to detect edges decreased at lower contrast. Our qualitative image analysis partly supports the latter finding as all three thoracic radiologists favoured higher ASiR-V levels, despite the fact that they perceived significantly more artefacts with ASiR-V. In terms of quantitative data, SNR/CNR and d’ both increased significantly with increasing ASiR-V levels to peak at 100%, with d’ remaining excellent for both high- and lower-contrast simulated pulmonary lesions, regardless of ASiR-V and dose levels.

At the time of writing, Euler *et al*. reported similar interesting results in a multiparametric phantom study^19^. The authors focused on the low-contrast detectability task, assessed by nine human observers for several conditions, and showed that 50% and 100% ASiR-V both significantly increased lesion detection. The spatial resolution of ASiR-V was found to be equivalent or slightly superior to that of FBP, except for low-contrast objects, which had lower resolution. However, they evaluated only two ASiR-V levels and did not investigate the effects of sharp kernels, nor compared their advanced physical metric results with clinical MDCT findings. Moreover, De Marco and Origgi recently reported in a further phantom study that the noise reduction potential of ASiR-V depended on the reconstruction section thickness and kernel, as well as the dose level below 2 mGy^40^. Interestingly, they found a dose-dependent behaviour of normalised NPS below 4 mGy for both smooth and sharp kernels. In particular, normalised NPS with the sharp kernel (bone, GE Healthcare) showed a negligible shift of their high-frequency peak with appearance of a second low-frequency peak with increasing ASiR-V levels. This intriguing discrepancy might be partly explained by differences inherent to bone and lung kernels. Unfortunately, the authors did not investigate the impact of ASiR-V on spatial resolution. On the other hand, Tang *et al*. reported in a recent study on regular-dose non-contrast chest CT that 60% ASiR-V yielded the highest overall image quality, while maintaining image sharpness compared with 40% ASiR, a statistical IR algorithm^18^. Similar to our qualitative results, diagnostic acceptability and artefact scores peaked at 60–70% ASiR-V. However, their analysis was limited by the use of conventional image quality metrics that did not account for the effects of IR techniques on noise texture, nor the contrast dependence of spatial resolution^23–27^.

Although our objective task-based assessment of image quality tends to favour high ASiR-V levels in regular-dose thoracic oncologic MDCT, using such high levels raises two main concerns. First, all three human observers perceived some loss of subtle image details (e.g. fine structures of secondary pulmonary lobules) and more IR-related artefacts with 100% ASiR-V. This somewhat IR-specific behaviour has been previously reported for almost all IR algorithms, with a typical blotchy or pixilated appearance of IR images^8,28,41^. These findings are only partly supported by our advanced physical metric analysis, showing slight shifts of NPS centroids with increasing ASiR-V levels, at both investigated dose levels. Second, for lower-contrast objects, the spatial resolution performances decreased with increasing ASiR-V levels, more markedly at reduced dose. This is in line with Paruccini *et al*. who recently showed using TTFs substantial changes in low-contrast spatial resolution with iDose and IMR (Philips Healthcare)^42^. Interestingly, they further reported that quantitative image blur (i.e. sharpness reduction for low-density details) increased with increasing levels of iDose and IMR. Furthermore, even though Yanagawa *et al*. recommended maximum ASiR levels for improving image quality on highly reduced-dose non-contrast pulmonary CT, they also found that excessive ASiR may obscure intralobular reticular opacities and peripheral vessels^43^. In contrast and intriguingly, Pontana *et al*. reported improved visualisation and conspicuity of CT features of systemic sclerosis-related interstitial lung disease (ground-glass opacities, reticulations, bronchiectasis, and/or bronchiolectasis) for reduced-dose images reconstructed with sinogram-affirmed IR (SAFIRE, Siemens Healthineers)^12^. However, this study was also limited by the inappropriate assessment of noise and spatial resolution properties with IR.

Since the release of IR techniques, thoracic MDCT image quality has been investigated extensively, with many studies reporting highly variable dose reduction potentials ranging from 13% to 76% for contrast-enhanced and 19% to as high as 99% for non-contrast CT, respectively^8–10^. However, such statements are futile if not related to the local baseline dose levels, which in turn vary with each clinical task. According to den Harder *et al*., reference dose levels with FBP in 24 contrast-enhanced chest CT studies varied widely from 1.5 to 21.8 mSv (using the 0.014 mSv/(mGy × cm) conversion factor)^9^. Furthermore, results from different studies are difficult to compare with one another because of the heterogeneity of the methods, large number of CT systems and protocols, various generations of IR algorithms, and different quantities and outcomes measured. Moreover, the vast majority of previous investigations ignored the non-linear properties of IR algorithms, making noise spatial-dependent and spatial resolution contrast-dependent^23–27^. Since conventional quantitative image quality metrics should be used with caution to assess IR images, most previous studies may have reported questionable data. In a review by Willemink *et al*., only one out of 33 chest CT studies with IR used NPS to fully characterise the noise properties, whereas none of them used TTFs to assess the contrast dependence of spatial resolution^10^. Finally, although human observer-based methods are popular, their qualitative and consequently subjective nature combined with the generally low number of observers and patients pose serious threats to their reliability and generalisability^26,44,45^. Our study certainly does not address all the issues mentioned above, yet it may pave the way for large-scale quantitative and automated task-based assessment of image quality with various IR techniques and MDCT protocols.

As mentioned earlier, conventional quantitative image quality metrics are ill-suited to IR algorithms^23–27^. In this study, we thus used advanced physical metrics that more accurately assess the impact of such algorithms on the noise, contrast, and spatial resolution properties, while further considering the clinical task. First, NPS allow for the full characterisation of overall noise by decomposing it as a function of its spatial frequency content, thereby reflecting noise texture. Noise texture has been reported as being influenced by both contrast and dose levels when using IR^35,46^. Intriguingly, we found that reducing the dose had no substantial effect on texture, while increasing the ASiR-V level generated only slight changes. Second, CNR, traditionally used as a surrogate for lesion detectability, assess contrast only at zero spatial frequency, thus allowing for significant bias with IR images, which have highly variable spatial frequency content. Third, MTFs evaluate spatial resolution in a high-contrast and low-noise environment, most often non-representative of clinical practice; they further do not consider its contrast and noise dependence with IR^23–27^. TTFs address these issues and should therefore be computed for various clinically relevant object-to-background contrast levels instead. Finally, even with NPS and TTFs, it remains challenging to correlate these metrics with diagnostic performance and ultimately clinical outcome, since image quality should always be optimised for a clinical task^47–49^; in our case, a detection task. Mathematical model observers, such as the NPWE, fulfil these needs by yielding d’ tailored to specific clinical tasks, depending on the levels of contrast, noise, and spatial resolution. The NPWE model observer has recently been updated for IR algorithms^34^, and validated for various high-contrast clinical tasks^35,50,51^. Lesion detectability strongly depends on the object’s size, shape, and contrast, as well as the noise and spatial resolution properties, all being concomitantly addressed by the NPWE. Additionally, d’ can be directly converted into AUCs, thereby providing an objective quantitative measure of diagnostic accuracy for a given detection task^52^.

Our study has a few limitations. First, it is a retrospective case series with a small sample size and only three human observers. However, a large number of advanced physical image quality metrics supplement these clinical data. Second, we only investigated the impact of a single partial model-based IR algorithm from a single CT manufacturer. Even though our results may be extrapolated to other partial model-based IR algorithms, a further comprehensive comparison across IR algorithms (including statistical and full model-based IR) and CT manufacturers and systems would be of great interest. However, this was beyond the scope of this initial investigation. Third, only two dose levels for two relatively simple yet specific clinical tasks were studied. We first favoured the detection task, yet other more complex tasks taking into account the lesion’s location, size, and shape are awaited. Fourth, we only quantified and characterised NPS for a phantom with uniform background. Even though Samei *et al*. and Håkansson *et al*. previously showed that the detection of subtle pulmonary nodules was limited by anatomical noise^53,54^, they also demonstrated that the same trends were observed using either uniform or textured backgrounds. In our two simulated clinical scenarios, the use of textured backgrounds would therefore have resulted in decreases in spatial resolution with shifts of spectral curve centroids toward lower spatial frequencies, thereby resulting in concomitant decreases in lesion detectability. However, detectability would have remained excellent for solid non-calcified nodules at both dose levels, and ground-glass opacities at regular dose. Finally, we did not directly compare the performance of human and mathematical model observers. However, the NPWE has recently been validated for similar high-contrast detection tasks^35,51^, while comparable trends have been reported with other model observers, such as the channelized Hotelling observer^19,35^.

In conclusion, while high ASiR-V levels (80%) are recommended for the detection of solid non-calcified nodules and ground-glass opacities in regular-dose thoracic oncologic MDCT, care must be taken because, for lower-contrast pulmonary lesions, high ASiR-V levels slightly change noise texture and substantially decrease spatial resolution, more markedly at reduced dose.

## Data Availability

The datasets generated and/or analysed during the current study are available from the corresponding author on reasonable request.
